# A Tunable Frequency Selective Rasorber with Broad Passband and Low Transmission Loss at X-Band

**DOI:** 10.3390/ma16175787

**Published:** 2023-08-24

**Authors:** Shengnan Shi, Zizhao Chai, Shan Zhang, Yanpeng Shi, Yifei Zhang

**Affiliations:** School of Microelectronics, Shandong University, Jinan 250100, China; 202112354@mail.sdu.edu.cn (S.S.); 202100400089@mail.sdu.edu.cn (Z.C.); 202132426@mail.sdu.edu.cn (S.Z.)

**Keywords:** frequency-selective surface (FSS), rasorber, tunable, varactor, passband

## Abstract

In this paper, we propose a dual-mode frequency selective rasorber (FSR) with tunable transmission and absorption windows at the X-band, which shows a broad passband in each transmission window. The proposed tunable FSR consists of a lossy absorption layer, a lossless transmission layer, and an air gap between them. The top frequency selective surface (FSS) layer is a cross-shaped meandering line with resistors and varactors for tunable absorption, and the bottom layer is a cross-shaped gap with varactors to achieve tunable bandpass. The equivalent circuit model (ECM) is investigated, and the 3D full wave simulation is performed. The results are based on simulations, and the simulation results show that the passband can be tuned from 12 to 8 GHz with an insertion loss between 0.5 and 1.4 dB by sweeping the capacitance of the varactors. The proposed design decreases the chances of detection by adversary devices and assures spectrum-safe communication, thereby creating new avenues for radar stealth and target concealment.

## 1. Introduction

An artificial electromagnetic periodic structure known as a frequency selective surface (FSS) [1] is capable of intentionally controlling the transmission and reflection of incoming electromagnetic waves. This property has made FSS structures increasingly popular in the area of antenna stealth technology [2,3,4,5,6,7,8]. FSS structures are designed to allow in-band signals to pass through with minimal insertion loss while significantly reflecting out-of-band signals, which is crucial for radar stealth applications. Recently, a frequency selective rasorber (FSR) with broadband absorption and low reflection has been suggested to solve this issue. This FSR generally consists of a low-loss passband along with one or two absorption bands [9]. Due to their unique properties, FSRs are considered essential for further improving the stealth performance. According to the authors’ best knowledge, the earliest concept of an FSR was proposed in 1995 [3], suggesting the use of a lossy dielectric layer on the FSS structure to absorb incident waves in a specific band. Later, Munk et al. [4] mentioned a conceptual design of an FSR without detailed structures and mechanisms for personal reasons. Subsequent works by various researchers [5,6,7,8,9] have explored different FSR designs, including hybrid designs with resistive metal cross oscillator or toroidal unit, which have applications in information security indoor wireless communication and anti-multipath systems. However, some of these designs suffer from significant insertion losses in the transmission band or relatively high transmission interpolation losses. In 2012, Costa [10,11] published an initial design of an FSR structure with excellent wave-transparent qualities at low frequencies and broadband absorption at high frequencies. The FSR design used a bandpass FSS operating at low frequencies and achieved minimal insertion loss in the transmission band. Analytical approaches based on equivalent circuit models have been employed for analyzing FSR performance. Subsequent research by Chen et al. in [12] proposed FSR designs with wave-transparent bands at high frequencies and broadband absorption at low frequencies. Introducing parallel resonant structures in the impedance plane unit further reduced the insertion losses and achieved low insertion loss transmission at high frequencies and broadband absorption at low frequencies in the work of Chen et al. [13,14] in 2017. These early FSR designs laid the groundwork for the theory and design of FSRs and have shown promising applications in wireless communications and radar stealth technology. As researchers continue to explore and refine FSR designs, the potential for improved stealth performance in various applications, including antenna systems, becomes increasingly evident.

In recent years, several designs for FSRs have emerged due to their selective transmission and strong stealth features. FSRs can be categorized into three groups according to the spectral positions of the passband and the absorption band: low-pass–high-absorption devices [15,16,17], high-pass–low-absorption devices [13,14,18], and bandpass devices with high- and low-absorption bands [19,20,21,22]. These designs are tailored to meet the requirements of antenna stealth in various radar systems. Two main types of FSRs have been proposed: the 3D model and the 2D model. The 3D model [23] offers high design freedom and structural strength but faces challenges related to complicated processing and high production costs. On the other hand, the 2D model [19,24,25], which consists of a three-layer structure, has minimal insertion loss in the top and bottom layers with no loss for electromagnetic waves in the center dielectric layer. Convoluted resonators [24], circular spiral resonators [18], and other structures have been proposed. However, further optimization is needed to address concerns related to the transmission bandwidth and structural complexity. Incorporating active devices like varactors into FSR designs allows for adaptive adjustments of the functional state for different electromagnetic environments [26,27,28,29,30,31,32,33,34,35,36,37,38,39,40,41]. Numerous models have been proposed based on this concept, offering tunable and customizable properties. For instance, Wu et al. [30] suggested an adjustable varactor sensor with three-layered metal layers, incorporating varactors and a bandpass frequency selective surface (FSS). However, the insertion loss of the FSR was relatively high, and the filtering responses of the absorption band were not reliable. Wang et al. [31] used a combination of a square ring array and an aggregate resistor as the components of a lossy layer to create an absorption band. While both layers were built using varactors, the transmission characteristics only satisfied unipolarization. Guo et al. [32] achieved an FSR using a folded crossed dipole combination of aggregate resistance and varactors to provide absorption characteristics. A passband lossless FSS was utilized with a sawtooth slot and varactors, but the tenability range was limited. Bakshi et al. [33,34] developed a multifunctional polarization-insensitive FSR by using lumped surface-mounted device (SMD) resistors and PIN diodes in the lossy layer. The lossless layer was created using J-C-based structure with varactors. However, the FSR could only be tuned to three discrete frequencies, limiting its continuous tuning capabilities. While these FSR designs show promising features, each has its limitations and potential for optimization. As researchers continue to explore and refine FSR designs, the potential for improved performance and applications in wireless communications and radar stealth technology becomes increasingly evident.

This paper introduces a novel tunable FSR designed for the X-band, featuring a tunable transparent window between two absorption bands. The tunable FSR was constructed by cascading a lossy FSS with a lossless FSS. The lossy FSS utilizes crosses combined with lumped resistors and varactors to achieve absorption characteristics, while the passband lossless FSS incorporates cross gaps and varactors. An equivalent circuit model (ECM) iwa developed to analyze the tunable operation in detail. The simulation results demonstrate that the center transmission frequency of the FSR can be continuously tuned from 12 to 8 GHz by adjusting the varactors from 0 to 0.04 pF. The insertion loss of the transmission window for the FSR is only 0.4 dB at 11 GHz, and it achieves a fractional bandwidth of 104.7% (4.76–15.22 GHz) with a −10 dB reflectivity under the normal incidence. Moreover, each transmission band has a relatively wide bandwidth, all greater than 1 GHz. The tunable FSR holds great potential for applications in ensuring the radiation performance of an antenna system in the in-band operating band for stealthy radomes. Additionally, it contributes significantly to the development of antenna stealth technology, making it a valuable contribution to the field.

## 2. Materials and Methods

**Figure 1:**
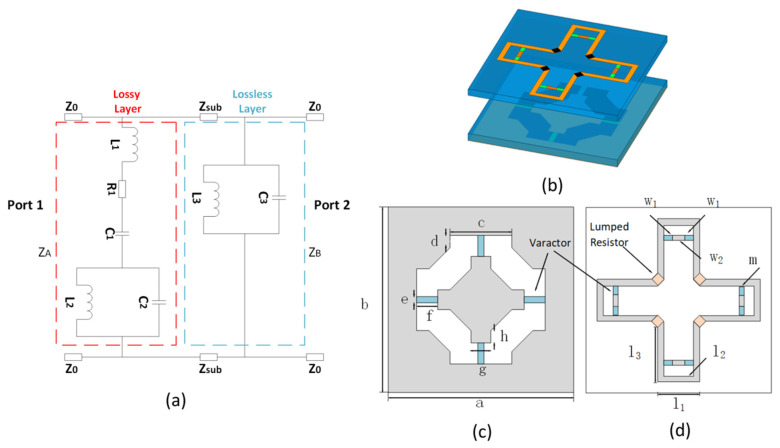
(**a**) General ECM of FSR, (**b**) unit cell of the proposed FSR (**c**) bottom view of the FSR (**d**) top view of the FSR.

As depicted in Figure 1a, the ECM of a tunable FSR can be separated into a lossy layer and a lossless layer, with $Z_{A}$ and $Z_{B}$ representing the impedances of the lossy layer and lossless layer, respectively. $Z_{0}$ and $Z_{sub}$ represent the characteristic impedance of the free and dielectric space, respectively. In this circuit, the transmission band is realized by the parallel LC in the lossy layer. The series RLC can simultaneously absorb out-of-band signals. The surface impedances of Figure 1 are as follows, according to [1]:

$$Z_{A}=R_{1}+j\frac{\left(\omega^{2}L_{1}C_{1}-1\right)\left(\omega^{2}L_{2}C_{2}-1\right)-\omega^{2}L_{2}C_{1}}{\omega C_{1}\left(\omega^{2}L_{2}C_{2}-1\right)}\;Z_{B}=\frac{j\omega L_{3}}{\omega^{2}L_{3}C_{3}-1},$$

where *ω* is the angular frequency.

For ECM, the passband is achieved to ensure that ${\;L}_{2}C_{2}=L_{3}C_{3}$; so, the resonance frequency of the circuits is

$$\omega_{p}=\frac{1}{\sqrt{L_{2}C_{2}}}=\frac{1}{\sqrt{L_{3}C_{3}}}.$$

According to the transmission line theory [28], the FSR can be analyzed as a two-port network, and the ABCD matrix of the network is

$$\left(\begin{array}{cc} A & B\\ C & D \end{array}\right)=\left(\begin{array}{cc} 1 & 0\\ \frac{1}{Z_{A}} & 1 \end{array}\right)\left(\begin{array}{cc} \cos\theta & jZ_{sub}\sin\theta \\ j\frac{\sin\theta }{Z_{sub}} & \cos\theta \end{array}\right)\left(\begin{array}{cc} 1 & 0\\ \frac{1}{Z_{B}} & 1 \end{array}\right)=\;\left(\begin{array}{cc} \cos\theta +j\frac{Z_{sub}}{Z_{B}}\sin\theta & jZ_{sub}\sin\theta \\ \left(\frac{1}{Z_{A}}+\frac{1}{Z_{B}}\right)\cos\theta +j\left(\frac{1}{Z_{sub}}+\frac{Z_{sub}}{Z_{A}Z_{B}}\right)\sin\theta & \cos\theta +j\frac{Z_{sub}}{Z_{A}}\sin\theta \end{array}\right),$$

where $\theta =\beta h=\frac{2\pi }{\lambda }h$, *h* is the electrical length or phase length of the spacer and is typically chosen to be approximately a quarter wavelength at the passband’s center.

To be more reasonable in design, foam sheet or air is usually used as the dielectric spacer, which means ${\;Z}_{0}=Z_{sub}$. The reflection coefficient $S_{11}$ and transmission coefficient $S_{21}$ can be expressed as:

$$S_{11}=\frac{-Z_{0}N+jZ_{0}\left(Z_{A}-Z_{0}-Z_{B}\right)\tan\theta }{2M+Z_{0}N+j\left(2M+{Z_{0}}^{2}+Z_{0}N\right)\tan\theta }$$

$$S_{21}=\frac{2M}{\left(2M+Z_{0}N\right)\cos\theta +j\left(2M+{Z_{0}}^{2}+Z_{0}N\right)\sin\theta },$$

where $M=Z_{A}Z_{B},\;\;N=Z_{A}+Z_{B}.$

For the transmission frequency ${\;f}_{T}$, it is important to ensure low insertion loss and wide bandwidth, so the impedances $Z_{A}$ and $Z_{B}$ should approach ∞. In this situation, $|S_{11}|=0$ and $|S_{21}|=1$, which means the incident wave moves from Port 1 to Port 2 without any loss. So, the center frequency of the transmission band is

$$f_{T}=f_{T_{1}}=f_{T_{2}}=\frac{1}{2\pi \sqrt{L_{2}C_{2}}}=\frac{1}{2\pi \sqrt{L_{3}C_{3}}}.$$

In addition, the absorption of the tunable FSR is calculates as:

$$A=1-{\left|S_{11}\right|}^{2}-{\left|S_{21}\right|}^{2},$$

which represents the percentage of energy absorbed by the lossy layer.

Figure 1b–d illustrates the proposed tunable FSR, which was derived from the designs that were described [35,36]. Within the structure, a lossy FSS layer is located on one side, and a lossless FSS layer is located on the other side of an air spacer that is a quarter of a wave in length. One may make a lossy layer by using either a resistive sheet or a resistive field-selective switch. Due to the fact that both layers are bandpass within the transmission band, the incoming electromagnetic wave is able to pass through the entire structure when it is being transmitted; however, it is able to be absorbed by the lossy material. The lossy layer of the tunable FSR presented in this research is designed to take the form of a cross-shaped structure that is loaded with four lumped resistors in each individual unit cell. The function of the lump resistors is to redirect the electromagnetic waves that are coming in from the outside. In addition, the absorptive FSS has eight varactors and four metal strips that have been shorted out. The increased frequency of the passband is the primary function that is served by the shorted metal strips. A bandpass FSS is configured in the form of a cross, and it serves as the lossless layer. The lossless FSS is modified by the addition of four varactors. On a substrate made of Arlon AD410 (FLEXcon, MA, USA), which has a relative permittivity of 4.1, the processing of the lossy layer takes place. The lossless layer is being processed on a Rogers RO4003 (ROGERS CORPORATION, CT, USA) with ɛr=3.55 and a 5 mm air gap in between the two substrates. The substrates of the lossy layer have a thickness of 0.7 mm, whereas the substrates of the lossless layer have a thickness of 0.8 mm. Based on existing models [38,39,40,41], the optimized parameters are:a=b=15 mm, c=5 mm, d=1 mm, e=0.5 mm, f=1.7 mm, g=1.6 mm, h=1 mm, $l_{1}=3.4\;mm$, $l_{2}=2.9\;mm$, $l_{3}=4.9\;mm$, $w_{1}=0.7\;mm$, $w_{2}=1\;mm$, m=0.5 mm, R=250 Ω.

**Figure 2:**
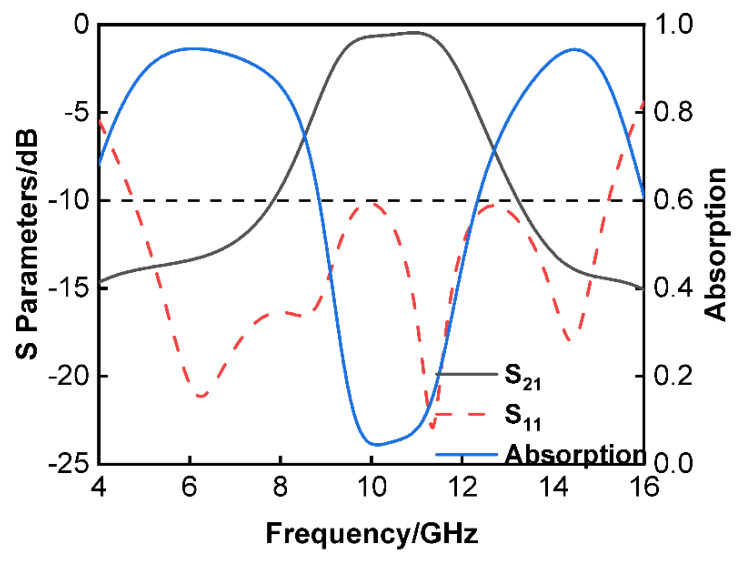
Reflection and transmission coefficients and absorption of the FSR calculated by HFSS.

Figure 2 illustrates the reflection and transmission coefficients as well as the absorption of the proposed tunable FSR that was based using the High-Frequency Structure Simulator (HFSS). According to the results of the simulation, the 3 dB transmission band of the proposed tunable FSR covered the frequency range of 9.06 to 11.98 GHz and had a relative bandwidth of 27.76%. At 11 GHz, an insertion loss of 0.4 dB was found to be the lowest possible value. In addition, the working bands with $S_{11}<-10\;dB$ were between 4.75 and 15.22 GHz. In other words, the proposed FSR has dual absorption bands at both 4.44–7.35 GHz and 12.76–15.42 GHz, and both of these bands have an AR of above 80%.

**Figure 3:**
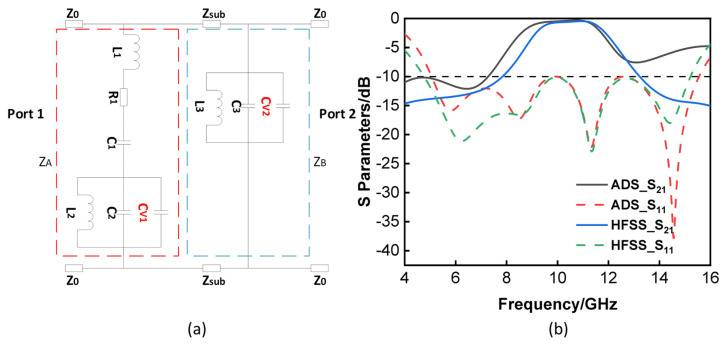
(**a**) The ECM of the tunable FSR. (**b**) Reflection and transmission coefficients of the FSR calculated by ADS and HFSS.

Eight varactors were built into each cell of the lossy layer to allow for fine control of the resonant frequency. The varactors are between the metal strip and the resonator cross. Varactors were similarly introduced to the lossless layer. On the lossy layer, a tunable frequency response is thus created by altering the equivalent capacitance between them. The same bias voltage can be used to generate the same tunable frequency response on the lossless layer with the correct design, leading to a fairly straightforward tuning process. As seen in Figure 3a, the varactors $C_{V1}$ and $C_{V2}$ can be used to determine the equivalent capacitances of resonators on both layers. We can obtain:

$$f_{T1}=\frac{1}{2\pi \sqrt{\left(C_{V1}+C_{2}\right)L_{2}}}=f_{T2}=\frac{1}{2\pi \sqrt{\left(C_{V2}+C_{3}\right)L_{3}}},$$

where $f_{T1}$ and $f_{T2}$ are variable capacitance resonator frequencies. Notably, to obtain a variable passband, the products of $L_{2}C_{2}$ and $L_{3}C_{3}$ must be synchronously variable.

Figure 3b demonstrates the reflection and transmission coefficients of the tunable FSR that were estimated using HFSS and Advanced Design System (ADS). The following were the values given to the parameters: $Z_{0}=Z_{sub}=377\;\Omega$, $R_{1}=250\;\Omega$, $C_{1}=0.042\;pF$, $C_{2}=0.0906\;pF$, $C_{3}=0.155\;pF$, $L_{1}=8.33\;nH$, $L_{2}=2.65\;nH$, $L_{3}=1.46\;nH$. The simulated 3 dB transmission band covered from 8.48 to 11.82 GHz (32.91%), and the working band with $S_{11}<-10\;dB$ was over 5–15.59 GHz. It is obvious that the curves corresponded with one another between the frequencies of 4 to 16 GHz. Due to the fact that the ADS is a simplified circuit model, it is unable to characterize the HFSS structure in its whole, particularly with regard to certain parasitic capacitances. There is a variation in high frequency between the two curves because the parasitic capacitance between the metallic vias that are vertical to the direction of the E-field was not taken into consideration. As the frequency of the signal rises, the influence of the parasitic capacitance becomes increasingly significant.

Table 1 contains the results of an in-depth comparison between the proposed FSR and other designs that have been reported. The purpose of this comparison was to show the improved performance of the planned structure. It is quite clear to see that a continuous adjustable range has been established and developed, and that this has been accomplished while keeping the insertion loss of our design to a minimum. At 11 GHz, the insertion loss that was modelled was less than 0.5 dB. The tuning range of this FSR was the largest of any FSR, including the whole X-band, and the working band of this FSR was additionally the widest of any FSR. This FSR had the widest tuning range. To put it another way, the fractional bandwidth for reflections less than −10 dB was calculated to be 104.7%, which is a considerable amount. In addition to this, the FSR is sensitive to both transverse electromagnetic waves (TE wave) and longitudinal electromagnetic waves (TM wave). When the angle of incidence was changed from 0° to 48°, the tunable FSR’s passband and absorption did not vary. This is true for both the transverse and the longitudinal polarizations. The findings of this paper also demonstrate that there was no solitary resonance present when an oblique occurrence was considered. It has been observed that the FSR has benefits such as low loss, a broad passband, and a wide operating band, illustrating the fact that it is superior to other FSR designs that are currently in use.

## 3. Results

**Figure 4:**
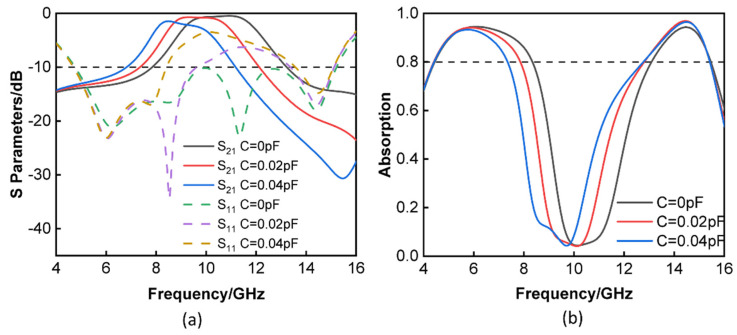
(**a**) Simulated S-parameters of the FSR at different capacitances of the varactor. (**b**) Simulated absorption of the FSR at different capacitances of the varactor.

It can be seen in Figure 4a that the value of the varactors may be changed from 0 to 0.04 pF, which results in a simultaneous shift in the center transmission frequency from 12 to 8 GHz. When the capacitor had a value of 0 pF, the insertion loss that was simulated was less than 0.5 dB at 11 GHz, and the fractional bandwidth for the reflection that was less than −10 dB was 104.7% (4.76–15.22 GHz). The proposed tunable FSR had a 3 dB transmission band that covered the frequency range of 8.49 to 10.98 GHz and had a relative bandwidth of 25.58% when the varactors were set to 0.02 pF. At 9.5 GHz, an insertion loss of just 0.7 dB was achieved. In the same way, the varactors were adjusted to measure 0.04 pF, and the 3 dB transmission band of the proposed tunable FSR spanned from 7.99 to 9.92 GHz and had a relative bandwidth of 21.55%. At a frequency of 8.5 GHz, an insertion loss of 1.4 dB was measured to be the lowest possible value. Only a slight increase in reflection occurred near the passbands as the capacitance rose. This was due to the transmission frequency mismatch between the several layers caused by the simultaneous capacitance adjustment. It is crucial to keep in mind that the varactor’s series resistance increased at lower bias voltages, which accounted for the increase in the insertion loss as the varactor’s size increased. The FSR may be absorbed at a variety of capacitances of the varactor, as in Figure 4b, which shows this phenomenon. The absorption rate was 0.08 at 11 GHz when the varactor was set to 0 pF, but it was over 80% from 4.44 to 8.34 GHz and 13.09 to 15.46 GHz. This is because the absorption rate increases with the frequency. The absorption rate was 0.06 at 9.5 GHz when the varactor was set to 0.02 pF; nevertheless, the range of 4.40 to 7.86 GHz and 12.80 to 15.42 GHz exhibited an absorption rate that was more than 80%. Changing the varactor value to 0.04 pF gave an absorbing rate of the order of 0.15 at 8.5 GHz. In comparison, the rate of absorption above 80% ranged from 4.41 at 7.35 GHz to 12.76 at 15.42 GHz. It demonstrates that the passband could be tuned by adjusting the varactors, which were responsible for adding the lossy layer and the lossless layer. As the capacitance increased and moved to lower frequencies, the 3 dB transmission band and absorption of the tunable FSR decreased.

**Figure 5:**
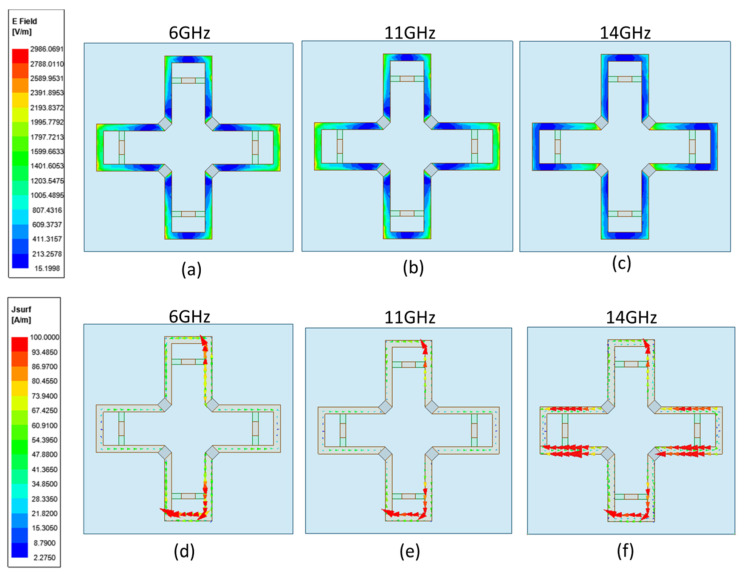
(**a**–**c**) E-field distribution under the different frequencies and (**d**–**f**) the surface current distribution under the different frequencies.

Figure 5a–f provide an illustration of the E-field and surface current distribution at various frequencies, which may be used to further investigate the mechanism behind the FSR. Resonance was produced by the lossy FSS that made up the top layer of the FSR, and the surface current was uniformly spread over the four metal patches. The lossy FSS of the lower layer only generated weak resonance on the upper metal patch, which mainly affected the low frequency transmission performance in the ECM; so, only the upper layer FSS was considered. There were substantial resonance currents on the higher and lower metal walls at the transmission frequency $f_{T}\;\left(11\;GHz\right)$, and the electric field was mostly on the left and right sides of the cross, as well as on the top and lower metal walls. In addition, huge currents flowed across the resistors that were aligned along the E-field; these resistors absorbed the incoming power at the two absorption bands. The distribution of the electric field and current at the lower absorption frequency $f_{L}=6\;GHz$ was similar to that of ${\;f}_{T}$. Because 6 GHz is low frequency, and the resonant wavelength is long, the electric field was mainly distributed on the upper and lower metal walls. At a higher absorption frequency $f_{H}=14\;GHz$, the electric field was mainly on the left and right sides of the resistance. Because of the strong resonance at 14 GHz, the closer to the resonant frequency, the greater the current in the circuit would be, so the current at 14 GHz became strong. The current was mainly distributed in the left and right metal walls of the cross shape, and some current also flowed through the upper and lower metal walls. The resistor current was minimal, resulting in excellent transmission and little insertion loss.

**Figure 6:**
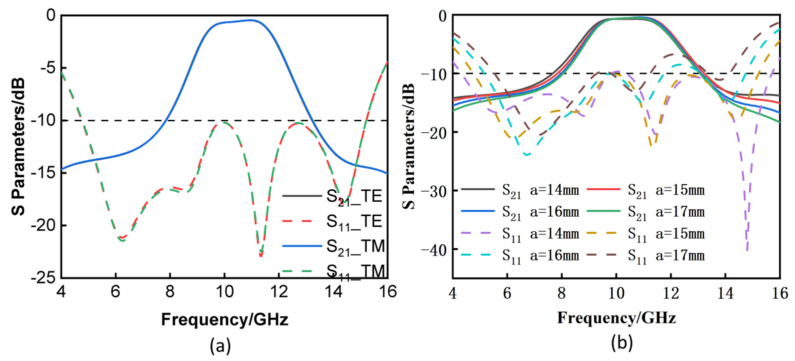
(**a**) Reflection/transmission coefficients of the FSR under TE and TM polarizations under normal incidence. (**b**) The effect of the length and width on the FSR.

**Figure 7:**
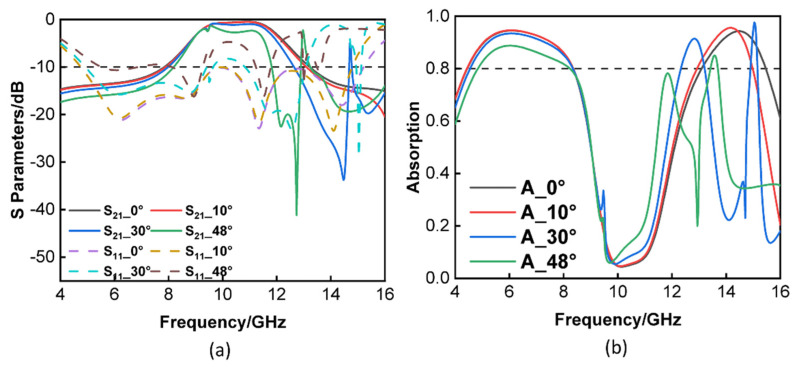

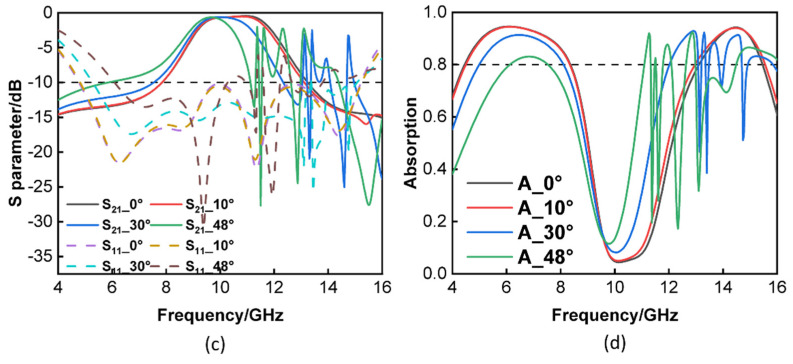
Reflection/transmission coefficients and absorption of the FSR under oblique incidence. (**a**,**b**) TE polarization. (**c**,**d**) TM polarization.

Figure 6a illustrates the reflection and transmission coefficients of the FSR under normal incidence for TE and TM polarizations. It is obvious to observe that the rates of reflection and transmission in both transverse electric and transverse magnetic polarizations were quite consistent with one another. The findings from the simulation indicated that the FSR design had dual polarization. Figure 6b verifies the effect of changing the length and width on the FSR. From the figure, it can be seen that when varying the length and width of the FSR, for the transmission wave, there was not much change. When a = 14 mm, the transmission band of the FSR ranged from 8.95 to 11.84 GHz, whereas when a = 17 mm, the −3 dB transmission band of the FSR went from 9.18 to 11.71 GHz. The only difference was that the working bandwidth of the FSR decreased slightly when a = 17 mm. The parameters were varied by 20%, and it can be seen that the device performance did not change much when the parameters were varied within a certain range. The performance of the suggested FSR when TE polarization was used was also taken into consideration and examined. When the angle was adjusted to 30°, as shown in Figure 7a, the transmission passband of the tunable FSR did not vary much; however, when the angle was changed to 48°, the transmission passband of the FSR was somewhat lowered; at this point, it was still capable of maintaining a transmission passband that was more than 1 GHz. Figure 7b shows that the impact of altering the angle was not very significant for a low frequency, but it was somewhat more significant for a high frequency; however, it was still possible to maintain 80% of the transmission efficiency. Additionally, when using TM polarization, the transmission passband of the tunable FSR did not change when the angle was changed from 0° to 10°; however, the transmission passband did experience a slight reduction when the angle was increased to 30° or 48°. When the angle was altered while the adjustable FSR was operating with TM polarization, it was easy to observe that the working band of the tunable FSR did not change much. In a similar manner, the angle had an effect on the absorption rate at high frequency when TM polarization was used. Figure 7 demonstrates conclusively that when the varactor was adjusted to either 0 or 0.8 pF, the transmission characteristics remained constant across a range that extended from 0 to 48°. When the incident angle was increased to 48°, the reflection coefficients remained stable at lower frequency bands. The rate of change in the absorption rate followed the same pattern as the changing trend in the reflection coefficient. As the incident angle increased from 0° to 48°, it was obvious that the transmission window could remain stable with a low insertion loss being experienced.

## 4. Conclusions

In order to study the operating principle of a tunable FSR, the equivalent circuit of an FSR was established first, and its equation was analyzed. Then, a lossy FSS and a lossless FSS were cascaded to create a tunable FSR, and its passband was modified using varactors. The lossless FSS is made up of cross-shaped slits with varactors, whereas the lossy FSS consists of varactors loaded with lumped resistors. The simulation results show that the transmission window can be tuned from 12 to 8 GHz when the varactor is tuned from 0 to 0.04 pF, with the insertion loss from 0.4 to 1.4 dB, along with low insertion loss. The tunable FSR operates over a wide bandwidth, because the reflectivity is kept within −10 dB in the frequency band of 4.76~15.22 GHz. And the bandwidth of each transmission band is guaranteed to be greater than 1 GHz, which is a relatively wide range compared to the FSRs that have been published. The proposed tunable FSR will find interesting applications in stealth and secure communication integration technologies due to the flexible passband and broadband absorption properties.

## Figures and Tables

**Table 1:** Comparisons between the tunable FSR and previous FSRs.

Ref.	Realization of Lossy FSS	Tunable Range (GHz)	Insertion Loss (dB)	$\mathrm{FBW}\;(\left|S_{11}\right|<-10\;dB)$	Oblique Performance	Polarization
[30]	metallic loops, extended metallic parts	1.6–3.3	3.4–9.3	95.9%	45°	dual
[31]	a square-loop structure	3.8–5.2	0.59–2.5	93.1%	30°	single
[32]	cross, meander lines	2.8–4.2	1.7–6.5	98.9%	30°	dual
[33]	a square loop, metallic strips	Discrete Freq. 4.01 GHz, 6.05 GHz, 13.1 GHz	0.55–1.01	-	30°	dual
[34]	a square loop, outer metallic patch	4.17–4.71	0.62–0.95	-	45°	dual
This work	a cross-shaped structure, metal strips	8–12	0.4–1.4	104.7%	48°	dual

## Data Availability

Not applicable.
